# Effect of High-Speed Sintering on the Optical Properties, Microstructure, and Phase Distribution of Multilayered Zirconia Stabilized with 5 mol% Yttria

**DOI:** 10.3390/ma16165570

**Published:** 2023-08-10

**Authors:** Mi-Hyang Cho, Hyo-Joung Seol

**Affiliations:** 1 Department of Dental Lab, Wonkwang Health Science University, Iksan-si 54538, Republic of Korea; milgong11@wu.ac.kr; 2 Department of Dental Materials, Dental and Life Science Institute, School of Dentistry, Pusan National University, Yangsan-si 50612, Republic of Korea

**Keywords:** high-speed sintering, translucency, opalescence, multilayered 5Y-zirconia, phase fraction

## Abstract

As dental 5 mol% yttria-stabilized (5Y-) zirconia demand high esthetics, it is necessary to clarify how the optical properties are affected by high-speed sintering, which is not yet fully understood. Our study aimed to investigate the effect of high-speed sintering on the translucency and opalescence parameters (TP and OP, respectively), as well as their related microstructure and phase distribution, using two types of multilayered 5Y-zirconia. Multilayered 5Y-zirconia (Cercon xt ML, Lava Esthetic) were cut layer-by-layer, followed by conventional and high-speed sintering. The TP and OP values were subsequently obtained using a spectrophotometer, and field emission scanning electron microscopy images were used to analyze the average grain size. The phase fractions were analyzed using X-ray diffraction. Regardless of the zirconia type, the TP was slightly lowered by high-speed sintering in all the layers except the dentin layer (DL) for Lava Esthetic (*p* < 0.05). The OP decreased by high-speed sintering in the DL for Cercon xt ML and in all the layers for Lava Esthetic (*p* < 0.05). The decrease in translucency after high-speed sintering was attributed to a decrease in the yttria-rich *t*’-phase with low tetragonality, along with an increase in the yttria-lean *t*-phase with high tetragonality.

## 1. Introduction

Natural human teeth possess various optical properties, such as translucency, opalescence, and fluorescence [1,2,3,4,5]. Zirconia, which is used as an esthetic restorative material in dentistry, has excellent strength and is esthetically superior to metals that have been predominantly used for dental prostheses in the past. Therefore, its use increased significantly in recent years. The translucency of zirconia can be improved by adjusting its yttria content. Conventional 3 mol% yttria-stabilized (3Y-) zirconia has low translucency; however, 4–6 mol% yttria-stabilized (4–6Y-) zirconia shows improved translucency with increasing yttria content [6,7,8,9]. This is because the optically birefringent tetragonal phase content decreases as the amount of yttria increases, while the cubic phase content, which is optically isotropic, increases [7]. Zirconia with improved translucency is being fabricated as monolithic esthetic prostheses that do not require porcelain veneers [10]. However, commercially available precolored monolithic zirconia still falls short of the translucency parameter (TP) of natural teeth [6], and further improvements are required.

Apart from being translucent, natural teeth also exhibit opalescent properties [4]. Owing to the opalescent effect, natural teeth appear bluish under reflected light and orange or brown under transmitted light [4]. The vitality of the restoration is increased by incorporating an opalescent effect into the restoration [11]. A study comparing the opalescence of 3Y-zirconia and ceria-stabilized zirconia/alumina nanocomposites with a fine two-phase structure found that the opalescence parameter (OP) of 3Y-zirconia increased linearly with increasing thickness [12]. However, a ceria-stabilized zirconia/alumina nanocomposite exhibited a significantly high OP value in the thin layer, which gradually decreased as the thickness increased [12]. The high OP value observed in the thin layer is attributed to the fact that the nanocomposite has a very fine two-phase structure, and there is a significant gap in the refractive index between the matrix and internal phase [12]. It has been shown in resin composites that when the TP values of the materials are very different, there is a strong correlation between the masking effect and the TP values [13,14]. However, when the TP values of the materials are similar, the opalescent properties can also affect the masking effect [13,15]. The OP value of human enamel is reportedly 19.8–27.6 at a thickness of approximately 1 mm [4]. However, the current OP value of dental zirconia does not reach that of human enamel [16].

Traditionally, the manufacture of zirconia prostheses involves a lengthy sintering process [17]. However, in response to the growing demand for single-day production of prosthetics, manufacturers introduced faster sintering schedules, such as speed sintering and, more recently, high-speed sintering, which achieve higher heating and cooling rates compared to that obtained with conventional sintering schedules [17,18]. During high-speed sintering, zirconia undergoes rapid temperature changes as it is rapidly heated to the sintering temperature and then rapidly cooled. The 3~6Y-zirconia, primarily used for dental prosthetics, undergoes phase transformations based on the temperature [19,20,21]. Scott presented an extensively accepted phase diagram for the ZrO_2_-Y_2_O_3_ system [21]. Based on Scott’s phase diagram, a phase transformation phenomenon occurring after the rapid cooling of yttria-stabilized zirconia (YSZ) at high temperatures was studied [22]. Consequently, YSZs with less than 7.5 mol% yttria decomposed into cubic and tetragonal phases by maintaining them at a temperature below 1425 °C, followed by dropping onto a brass plate to rapidly cool to room temperature within 10–20 s. However, when YSZs were rapidly cooled from temperatures of 1425 °C or higher, a metastable tetragonal phase was formed instead of a cubic phase [22]. The high-speed sintering schedule for YSZs in dental prostheses allows zirconia to be sintered at 1500 °C or higher, followed by rapid cooling at a rate of approximately 120 °C/min or higher. Although this cooling rate was slower than that obtained by dropping onto a brass plate, it was still significantly higher than the cooling rate of a conventional sintering schedule, which is approximately 10 °C/min. Therefore, the resulting phases may vary from those obtained from conventional sintering [18,23], and the various properties of dental zirconia obtained through high-speed sintering may not be equivalent to those of zirconia sintered with conventional sintering schedules.

Recently, advances have been made in the production of zirconia with improved translucency and a multilayered structure, as well as the investigations about the multilayered ceramics [24]. The multilayered zirconia materials are commonly accompanied by high-speed sintering schedules provided by the manufacturers. As these high-speed sintering schedules become more widely available, research on the effects of the sintering speed on the optical and mechanical properties of zirconia is being actively conducted. Conventional, speed, and high-speed sintering of Katana superior translucent multi layered, 5Y-zirconia (STML) showed that speed sintering did not result in any phase change, whereas high-speed sintering showed a decrease in the cubic phase and an increase in the tetragonal phase [23]. Consequently, although high-speed sintering did not significantly affect the TP of Katana STML (5Y-zirconia), it resulted in an increase in the biaxial flexural strength [23]. Another study of high-speed sintered Katana STML under slightly different sintering conditions reported no significant changes in the phase fraction or TP [25]. However, the fracture toughness decreased with high-speed sintering [25]. In contrast, for non-multilayered 5Y-zirconia, such as Zpex Smile and Prettau Anterior, high-speed sintering resulted in a significant decrease in the TP and flexural strength [26,27]. Microstructural observations revealed an increase in the grain size and formation of internal pores by high-speed sintering [26,27]. In addition, changes in the crystal phase fraction were observed in Zpex Smile [27]. Therefore, the impact of high-speed sintering on the characteristics of 5Y-zirconia can vary based on the product. Thus, further research is warranted to understand how high-speed sintering affects the optical properties of dental zirconia, which requires good esthetics. In particular, for zirconia products with a multilayered structure, analyzing the changes in the optical properties of each layer would provide a clearer understanding of the impact of high-speed sintering on the optical properties [7,18]. This study aimed to investigate the effects of high-speed sintering on the translucency and opalescence, as well as the related phase fraction and microstructure, of two types of multilayered 5Y-zirconia. The null hypothesis was that high-speed sintering would not have any impact on the optical properties, phase fractions, or microstructures of the two types of 5Y-zirconia.

## 2. Materials and Methods

### 2.1. Specimen Preparation

**Figure 1:**
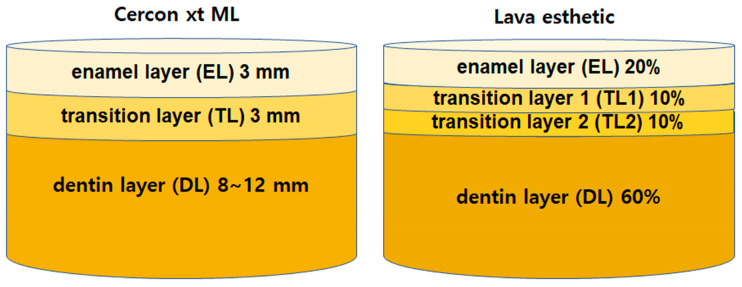
Layer fraction in a section of Cercon xt ML and Lava esthetics specified by the manufacturers.

The zirconia (Table 1) used in this study, Cercon xt ML (Dentsply Sirona, Charlotte, NC, USA) and Lava Esthetic (3M, St. Paul, MN, USA), were both precolored (A2 shade) 5Y-zirconia. Both zirconia samples had a multilayered structure, and a cutting machine was used (Accutom-100, Struers, Copenhagen, Denmark) to cut the specimens according to the layer fraction specified by the manufacturers (Figure 1). The upper and lower surfaces of the cut specimens were then sequentially dry polished with SiC-abrasive papers of 800, 1200, 2000, 3000, and 5000 grit sizes to reach a thickness of 1.25 mm for each layer (n = 5/layer), which was confirmed using micrometers (MDC-25PX, Mitutoyo, Kanagawa, Japan). Thereafter, sintering was carried out using a furnace (inLab Profire, Dentsply Sirona, Charlotte, NC, USA) following the manufacturer’s instructions for conventional and high-speed sintering (Table 2). Because the Lava Esthetic did not provide a high-speed sintering schedule, the schedule used for Cercon xt ML was applied for high-speed sintering of Lava Esthetic. The final dimensions of the sintered specimens were 10.0 × 10.0 × 1.016 mm^3^ (±0.008).

### 2.2. Optical Properties Analysis

A spectrophotometer (CM-3600d, Konica Minolta Sensing Inc, Osaka, Japan) was used to analyze the optical properties based on the ISO/TR 28642 [28]. Measurements were conducted using a CIE standard illuminant D65 and a 2-degree standard observer. The measurements were conducted three times, with a measurement interval of 10 nm ranging from 360 to 740 nm (n = 5/group). Spectral reflectance measurements were performed in the reflectance mode, including ultraviolet, with white (a* = −0.09, b* = 0.97, L* = 99.07) and black (a* = 0.71, b* = −0.41, L* = 9.09) backgrounds. For the transmittance measurements, the specimens were placed at the entrance hole of an integrating sphere and measured in the transmittance mode. The color coordinates for lightness (L*), red-green chromaticity index (a*), and yellow-blue chromaticity index (b*) were determined using the Spectra-Magic software (version 2.02, Konica Minolta Sensing Inc, Osaka, Japan).

To obtain TP of the specimen, the CIEDE2000 color difference (ΔE_00_) was calculated with the values obtained from reflectance measurements against white and black backgrounds using the following equation [29,30]:

(1) $$\mathrm{CIEDE}2000\;(\Delta E_{00})={\left[{\left(\frac{\Delta L^{\prime }}{K_{L}S_{L}}\right)}^{2}+{\left(\frac{\Delta C^{\prime }}{K_{C}S_{C}}\right)}^{2}+{\left(\frac{\Delta H^{\prime }}{K_{H}S_{H}}\right)}^{2}+R_{T}\left(\frac{\Delta C^{\prime }}{K_{C}S_{C}}\right)\left(\frac{\Delta H^{\prime }}{K_{H}S_{H}}\right)\right]}^{1/2}$$

where ΔL’, ΔC’, and ΔH’ are the differences in the lightness, chroma, and hue of a given set of samples, respectively.

K_L_, K_C_, and K_H_ are parametric factors used to compensate for the mismatch in the experimental conditions; they were fixed at 1 in the present study [30,31]. S_L_, S_C_, and S_H_ correspond to the weighting functions for lightness, chroma, and hue, respectively. R_T_ represents the rotation function, which is utilized to adjust for the interaction between the differences in chroma and hue in the blue region. To evaluate the ΔE_00_ value, perceptibility thresholds of 50:50% (ΔE_00_ = 1.30 and 1.25 for the fitted S-shape curves and Takagi-Sugeno-Kang (TSK) Fuzzy Approximation, respectively) and acceptability thresholds of 50:50% (ΔE_00_ = 2.25 and 2.23 for the fitted S-shape curves and TSK Fuzzy Approximation, respectively) were used [31].

OP was obtained using the following equation [4,13]

OP = [(a*_T_ − a*_R_)^2^ + (b*_T_ − b*_R_)^2^]^1/2^ (2)

Here, subscript T represents the transmitted light and subscript R represents the reflected light measured against a black background [4]. Δa* and Δb*, which are the differences in the red-green (a*) and yellow-blue (b*) coordinates between the color observed in reflected and the transmitted light, respectively, were determined from (a*_T_ − a*_R_) and (b*_T_ − b*_R_), respectively.

### 2.3. Field Emission Scanning Electron Microscopy (FE-SEM) Analysis

To analyze the microstructures of the specimens (n = 2/group), FE-SEM examination (JSM-7200F, Jeol, Akishima, Japan) was performed. The accelerating voltage was set to 10 kV. Prior to the analysis, a platinum sputter coating was applied to the specimens for 90 s. The obtained FE-SEM images were used to analyze the crystal size of each layer based on the standard ASTM E112. The linear intercept method [7,32] was employed for this purpose, and more than 1000 crystals were analyzed for each layer. Image J Software (version 1.53e, National Institutes of Health, Bethesda, Rockville, MD, USA) was used for the analysis, and the grain size (D) was determined by applying the following equation:

D = 1.56C/MN(3)

where C denotes the line length used, M is the magnification of the microscopic image, and N is the number of intercepts used. The coefficient of correction was 1.56 [7,9].

### 2.4. X-ray Diffraction (XRD) Study

For high-resolution XRD analysis, Ni-filtered CuKα radiation was used (n = 1/group, X’Pert3 powder, PANalytical, Amsterdam, Netherlands) in the range of 25–90 degrees (2θ). The step size used for the analysis was 0.013 degrees (2θ), and the tube current and voltage were 30 mA and 40 kV, respectively. XRD patterns were subjected to Rietveld analysis using Topas Academic software V 7.21 (Bruker AXS, Karlsruhe, Germany). The fractions of the tetragonal, cubic, and monoclinic phases were determined. The space groups of the tetragonal, cubic, and monoclinic phases were P4_2_/nmc, Fm3m, and P2_1_/c, respectively [33]. The standard Crystallographic Information files (CIF) were obtained from the Crystallography Open Database (COD), and the CIF for tetragonal, cubic, and monoclinic phases were obtained from Howard et al. [34], Lamas and Walsoe de Reca [35], and Smith et al. [36]. The refinement quality was maintained by controlling the Rwp value to be below 5%. To calculate the Y_2_O_3_ (mol%) content based on the lattice parameters (*a*,*c*) of the tetragonal phase, the following equations were used [19,27]:

(4) $${\mathrm{YO}}_{1.5}\;(\mathrm{mol}\%)=\frac{1.0223-c/a\sqrt{2}}{0.001319}$$

(5) $$Y_{2}O_{3}\;(\mathrm{mol}\%)=\frac{{YO}_{1.5}(mol\%)/100}{2-{YO}_{1.5}(mol\%)/100}\cdot 100$$

### 2.5. Statistical Analysis

Statistical analysis was conducted using statistical software SPSS 25.0 (Statistical Product and Service Solutions 25.0, IBM Co., Armonk, NY, USA) and a significance level of 0.05 was set. The data’s normality was assessed using the Shapiro–Wilk test. To analyze the effects of zirconia type, sintering speed, and each layer of zirconia on TP, OP, Δa*, and Δb*, a three-way analysis of variance (ANOVA) followed by a post hoc Tukey’s HSD test was conducted. Because the grain size did not meet the assumption of normality, non-parametric methods (Kruskal–Wallis H test followed by the Bonferroni–Dunn post hoc test) were used. For grain size comparisons between the layers, the Mann–Whitney U test was employed.

## 3. Results

### 3.1. Translucency Parameter (TP)

The results of the three-way ANOVA (Table 3) suggested that the type of zirconia used did not have a significant effect on the TP. However, the sintering speed (*p* < 0.001) and layer (*p* < 0.001) had a significant effect on the TP. An interaction was observed between the type of zirconia used and the layer (*p* < 0.001).

High-speed sintering (Table 4) resulted in a lower TP in all the layers for both types of zirconia, except for the dentin layer (DL) of the Lava Esthetic (*p* < 0.05). Cercon x t ML showed a slight increase in TP from the enamel layer (EL) to the DL at both sintering speeds (*p* < 0.05). Conventionally sintered Lava Esthetic showed a slight decrease in the TP from EL to DL (*p* < 0.05). The TP values were not significantly different among the layers for the high-speed-sintered Lava Esthetic. When the sintering speed was the same, the TP values were not significantly different between the two zirconia samples (except for the EL and conventionally sintered DL, *p* < 0.05).

### 3.2. Opalescence Parameter (OP)

The findings derived from the three-way ANOVA (Table 5) suggested that the type of zirconia (*p* = 0.002), sintering speed (*p* < 0.001), and layer (*p* < 0.001) had a significant effect on the OP. An interaction was observed between the type of zirconia used and sintering speed (*p* = 0.004), as well as between the sintering speed and layer (*p* = 0.018).

High-speed sintering decreased the OP only in the DL for Cercon xt ML and in all the layers for Lava Esthetic (Table 6). Both Cercon xt ML and Lava Esthetic demonstrated an increase in the OP from EL to DL, regardless of the sintering speed (Table 6, *p* < 0.05). The OP values were not significantly different between the two zirconia when conventionally sintered (*p* < 0.05). However, with high-speed sintering, Lava Esthetic demonstrated lower OP values than Cercon xt ML in the EL and transition2 (T2) layers (*p* < 0.05).

High-speed sintering decreased Δa* in all the layers for both zirconia types; however, Δb* decreased only in the DL for Cercon xt ML and in all the layers for Lava Esthetic (Table 7, *p* < 0.05). Both Δa* and Δb* increased from EL to DL, irrespective of the type of zirconia and sintering speed. The increase in Δa* was minimal, whereas the increase in Δb* was more noticeable.

### 3.3. Microstructure

**Figure 2:**
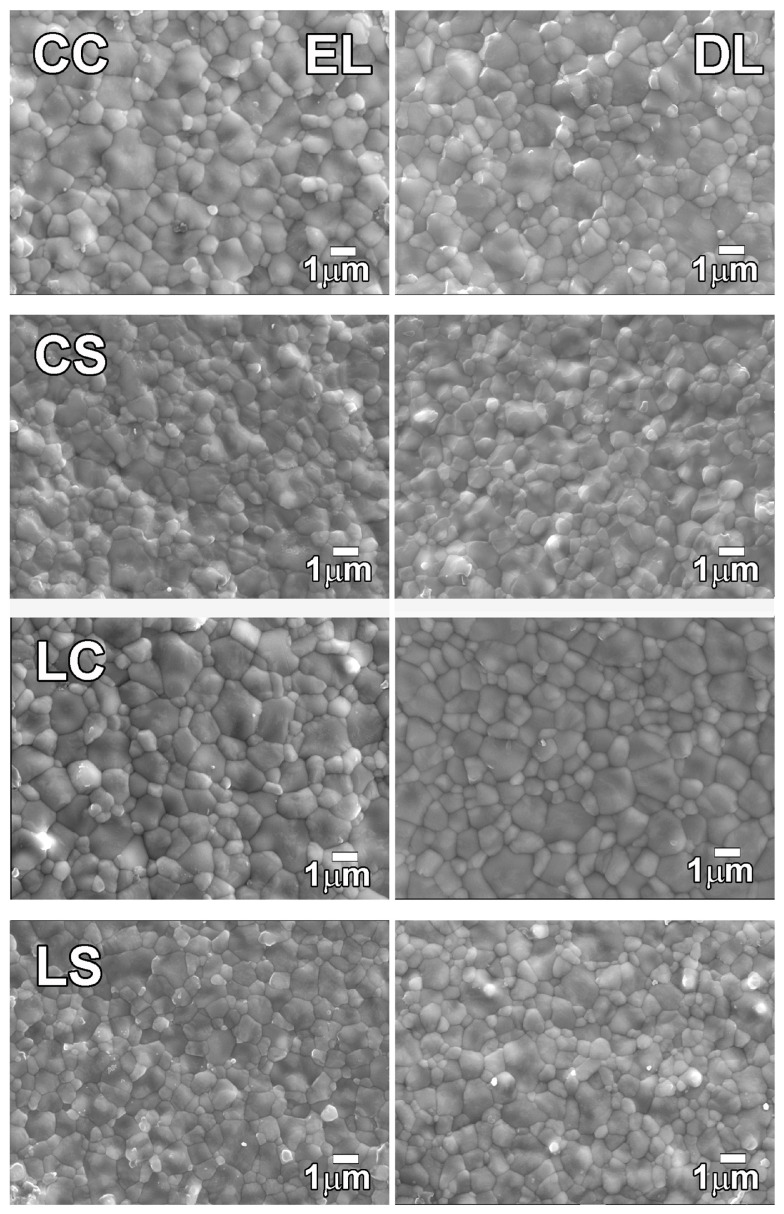
Microstructure for each group of the Cercon xt ML and Lava esthetic specimens (8000×). CC, conventionally sintered Cercon xt ML; CS, high-speed sintered Cercon xt ML; LC, conventionally sintered Lava esthetic; LS, high-speed sintered Lava esthetic; EL, enamel layer; DL, dentin layer.

SEM observations revealed that both types of zirconia exhibited equiaxed crystal structures with a mixture of larger and smaller crystals (Figure 2). No apparent difference was observed in the microstructures of the EL and DL in any of the specimens. Crystal size analysis of the EL and DL (Table 8) showed that only Lava Esthetic demonstrated a decrease in the crystal size after high-speed sintering (*p* < 0.05); however, the difference was minimal. Neither type of zirconia exhibited significant differences in the crystal size between the EL and DL. The crystal size of all the specimens was approximately 1–1.2 μm.

### 3.4. XRD Analysis

**Figure 3:**
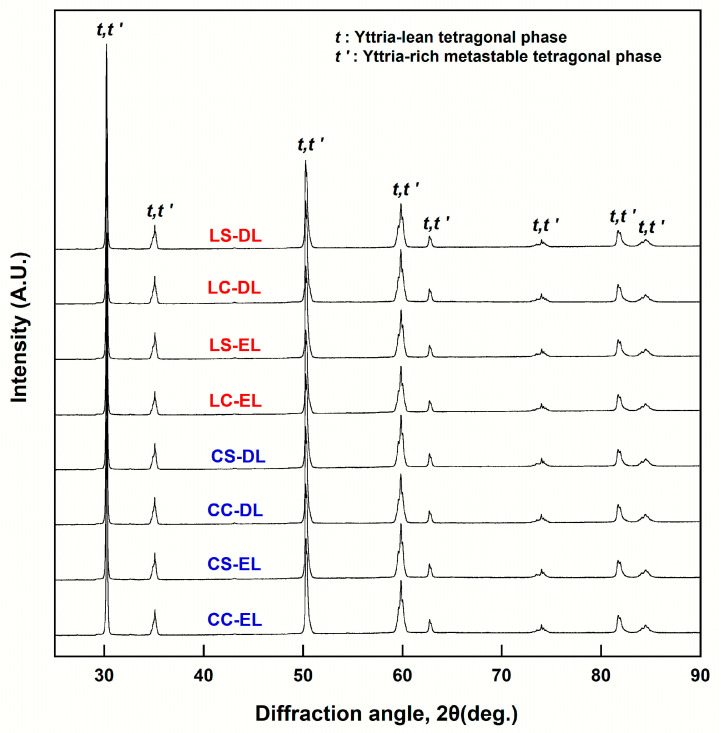
X-ray diffraction patterns for each group of the Cercon xt ML and Lava esthetic specimens. CC, conventionally sintered Cercon xt ML; CS, high-speed sintered Cercon xt ML; LC, conventionally sintered Lava esthetic; LS, high-speed sintered Lava esthetic; EL, enamel layer; DL, dentin layer.

An analysis was performed on the XRD patterns (Figure 3) of the sintered EL and DL specimens (Table 9). Both the zirconia samples were composed of two phases, a metastable tetragonal (*t*’) and tetragonal (*t*) phase, at both sintering speeds. Both types of zirconia exhibited a slight reduction in the *t*’-phase and a slight increase in the *t*-phase in both the EL and DL after high-speed sintering. The difference in the phase content between the EL and DL was small. Irrespective of the type of zirconia used and the sintering speed, the axial ratio (tetragonality, *c/a*√2) of the *t*’-phase was ~1.005, while the axial ratio of the *t*-phase was ~1.015. Additionally, the *t*’-phase exhibited a higher yttria content compared to that of the *t*-phase.

## 4. Discussion

This study was conducted to investigate the differences in the optical properties, microstructure, and phase fraction between high-speed sintered and conventionally sintered multilayered 5Y-zirconia. The experimental results provided partial evidence against the null hypothesis, suggesting that high-speed sintering has an effect on the optical properties, microstructure, and phase fraction of the zirconia used. The TP value slightly decreased in all the layers (except the DL of Lava Esthetic) by high-speed sintering, regardless of the zirconia type (*p* < 0.05). For Katana STML (5Y), for which the manufacturer provides a high-speed sintering schedule, the TP value did not change with high-speed sintering [23,25,26]. However, for the Prettau Anterior and Zpex Smile, for which the manufacturer does not provide a high-speed sintering schedule, the application of high-speed sintering results in the formation of internal porosities, leading to a significant decrease in translucency by approximately 50% [26]. Lava Esthetic, which does not have a high-speed sintering schedule, and Cercon xt ML, which has a high-speed sintering schedule, exhibited a slight decrease in translucency due to high-speed sintering. However, the variation in TP caused by high-speed sintering was below the perceptibility thresholds by CIEDE2000 (ΔE_00_ = 1.30) [31]. Therefore, the decrease in the TP caused by high-speed sintering in these two types of zirconia is not clinically significant. Similarly, in a study using 4Y- and 6Y-zirconia for high-speed sintering, the TP of some layers increased in 4Y-zirconia but decreased in 6Y-zirconia. Nevertheless, the variation in the TP was below the perceptibility thresholds by CIEDE2000 [18]. However, the final translucency was mainly affected by the substrate as well as the type of material and cement in the final esthetic outcome of ceramic restorations [14].

Lava Esthetic showed a slight decrease in TP from the EL to DL (*p* < 0.05) after conventional sintering, while no significant interlayer TP difference was observed following high-speed sintering. In contrast, Cercon xt ML showed a slight increase in TP from the EL to DL, regardless of the sintering speed (*p* < 0.05). Therefore, the changes in translucency from the EL to DL varied according to the type of zirconia and sintering speed; however, the TP changes from the EL to DL were lower than the perceptibility thresholds of CIEDE2000 (ΔE_00_ = 1.30) [31]. The multilayered Katana STML (5Y, A2 shade) and Katana ML (5.5Y, A1.5-2 shade) were reported to show a decrease in the translucency from the EL to DL [16,37], which was caused by a greater discrepancy in the pigmentation or changes in the pigmentation from the EL to DL [37]. As no apparent difference in the crystal size and phase fraction was observed between the EL and DL for both zirconia types in this study, the TP changes from the EL to DL were considered to be mainly attributed to the difference in the zirconia composition and variations in pigment type and quantity [6,16].

Irrespective of the sintering speed, the difference in the TP values between Lava Esthetic and Cercon xt ML was below the perceptibility thresholds by CIEDE2000 (ΔE_00_ = 1.30) [31]. The TP values of the EL ranged from 8.17 to 9.33, and the TP values of the DL ranged from 8.83 to 9.60 at a thickness of 1 mm following conventional and high-speed sintering. In yttria-stabilized zirconia (YSZ), the TP exhibited an exponential decrease as the thickness increased [12,38]. For A2 shade zirconia (4Y), the TP value was reported to exponentially decrease from 12.54 to 5.4 as the thickness increased from 1 mm to 2 mm [39]. The TP values of natural human dentin are approximately 7 and 21 for the incisors and molars, respectively, at a thickness of 2 mm [1]. Considering the decrease in the TP with increasing thickness, the TP values in this study were lower than those obtained for human incisor dentin. This finding is consistent with previous research [6]. In the case of one or A2-shaded resin-based composites, the TP values were reportedly much higher (15–25 at 1 mm thickness) than those in this study [40].

Natural human teeth exhibit opalescent properties in addition to their translucent properties [4]. Owing to the opalescent effect, natural teeth appear bluish under reflected light and orange or brown under transmitted light [4]. After high-speed sintering, Cercon xt ML demonstrated a minor decrease in the OP only in the DL, whereas Lava Esthetic showed a slight decrease in the OP in all the layers (*p* < 0.05). A slight decrease in the OP after high-speed sintering was also reported for 4Y- and 6Y-zirconia, and their OP values were similar to those of the 5Y-zirconia in this study [18]. The OP was calculated based on Δa* and Δb*, which are the differences in the red-green coordinate (a*) and yellow-blue coordinate (b*) between the color observed in the reflected and transmitted light, respectively. Differences in lightness (ΔL*) resulting from the reflected and transmitted colors were reported to show limited correlation with OP [4] and, thus, ΔL* was excluded from the equation for calculating OP [4,13]. Δa* decreased in all the layers after high-speed sintering for both types of zirconia. However, Δb* decreased only in the DL for Cercon xt ML and in all the layers for Lava, which was consistent with the change in the OP. Human and bovine enamel also demonstrated a high correlation between OP and Δb* [4]. To facilitate a comparison with the optical properties of natural human teeth, A2-shaded zirconia, which is widely used, was selected for this study. The OP values of the human EL (with a thickness of ~1 mm) reportedly range from 19.8 to 27.6 [4]. The measured OP values in this study were found to be lower than those of natural human teeth. Furthermore, the OP values decreased with high-speed sintering. However, considering that the OP values of human teeth are higher and exhibit a wider range than those obtained in this study, the decrease in the OP values due to high-speed sintering was not considered clinically significant. For commercial resin composites, the OP values reportedly range from 5.7 to 23.7 (at a thickness of 1 mm), varying based on the resin brand and shade [41].

The two types of zirconia used in this study were multilayered zirconia with a color gradient. The OP values increased from the EL to DL, regardless of the sintering speed, for both types of zirconia (*p* < 0.05). This trend was also reported for 4Y- and 6Y-zirconia [18]. Upon analyzing the changes in Δa* and Δb* related to the OP, the increase in Δa* from the EL to DL was minimal, whereas the increase in Δb* was greater for all the specimens. This increase in Δb* contributed to a significant increase in the OP from the EL to DL. In YSZ, the OP value increases with increasing thickness [12,39,42]. In colored zirconia, the increase in the OP value with increasing thickness was greater than that in uncolored zirconia [39,42].

Minimal or no change in the grain size was observed for both 5Y-zirconia after high-speed sintering, as it was reported in multi-layered 4Y- and 6Y-zirconia with grain sizes of approximately 0.5 µm and 2 µm, respectively [18]. The obtained grain size in this study was approximately 1–1.2 µm, which was close to the average grain size of the reported 4Y- and 6Y-zirconia [18]. This value was also close to that obtained from the conventionally sintered Katana STML (5Y, 1.11 µm) [43]. In contrast, for high-speed sintered Katana STML, contradictory results regarding the change in grain size have been reported, indicating both an increase and decrease due to high-speed sintering [23,25]. These studies used the same sintering furnace, zirconia, and sintering temperature, but different heating rates, cooling rates, and holding times during sintering [23,25]. As the zirconia grain size is influenced by conditions used for sintering, such as sintering temperature and holding time, the conflicting results were thought to be attributed to the differences in the sintering conditions [44].

Based on the Rietveld analysis of the XRD data, both types of zirconia showed a slight decrease in the metastable *t*’-phase and slight increase in the *t*-phase in both the EL and DL by high-speed sintering. The DL of the Lava Esthetic, which did not show a significant change in translucency after high-speed sintering, exhibited the least variation in the phase content. Irrespective of the type of zirconia used and the sintering speed, the axial ratio (tetragonality, *c/a*√2) of the *t*’-phase was approximately 1.005, while the axial ratio of the *t*-phase was approximately 1.015. Therefore, the *t*-phase exhibited higher tetragonality than the *t*’-phase. These findings are consistent with those reported in the literature [19,27]. The cubic phase is optically isotropic, whereas the *t*-phase has tetragonality, which causes optical birefringence and decreases translucency. The metastable *t*’-phase has higher yttria content than does the *t*-phase [19,27], as observed in this study. This causes a slight decrease in the axial ratio (tetragonality) in the metastable *t*’-phase, resulting in higher translucency compared to that in the *t*-phase [33]. Based on these findings, the decrease in translucency after high-speed sintering observed in this study was attributed to a decrease in the yttria-rich *t*’-phase with low tetragonality, along with an increase in the yttria-lean *t*-phase with high tetragonality. Similarly, in a study where Katana STML (5Y) was high-speed-sintered at 1560 °C with a holding time of 6.9 min, a decrease in the cubic phase was observed along with an increase in the tetragonal phase with high tetragonality [23]. In a study using a high-speed-sintered Zpex Smile (5Y), the *t*-phase decreased while the *t*’-phase increased, contradicting the findings of our study [27]. However, despite the increase in the *t*’-phase, the translucency decreased owing to the increased number of residual pores caused by high-speed sintering, as well as the decrease in flexural strength [27]. Thus, it can be concluded that the changes in the phase fraction due to high-speed sintering of 5Y-zirconia vary based on the zirconia composition and sintering conditions.

This study had certain limitations that should be acknowledged. First, the study used only two types of zirconia, which may not fully represent the entire range of available zirconia materials. Consequently, the findings of this study may not be applicable to other types of zirconia. Additionally, variations in the high-speed sintering conditions were limited. There was also the lack of aging for the long-term evaluation of the investigated variables. Consequently, the generalizability of the study’s conclusions may be restricted to the specific conditions investigated. Thus, different results can be obtained using zirconia from different manufacturers or by altering the protocol for high-speed sintering. To obtain more conclusive results, further studies are needed. 

## 5. Conclusions

Regardless of the type of zirconia, high-speed sintering resulted in a slight decrease in the TP in all layers (except the DL for Lava Esthetic). After high-speed sintering, the OP decreased only in the DL for Cercon xt ML, whereas for Lava Esthetic, it decreased in all the layers. These changes in the OP corresponded to the changes in Δb*. The minor reduction in translucency owing to high-speed sintering was attributed to a slight decrease in the yttria-rich *t*’-phase, accompanied by a slight increase in the yttria-lean *t*-phase.

## Figures and Tables

**Table 1:** Composition of the utilized materials.

Zirconia	Type	Chemical Composition (wt%)			
		ZrO_2_	HfO_2_	Y_2_O_3_	Al_2_O_3_ + SiO_2_ + Others
Cercon xt ML	5Y-zirconia	>86	<3	9	<2
Lava Esthetic	5Y-zirconia	Unknown			

**Table 2:** Sintering schedules of the used materials.

Sintering Speed	Zirconia	Code	Stage	Rate of Heating and Cooling (°C/min)	Temp. (°C)	Holding Time (min)
Conventional	Cercon xt ML	CC	1	22	880	0
			2	11	1500	135
			3	99	300	0
			4	25	50	0
	Lava Esthetic	LC	1	20	800	0
			2	10	1500	120
			3	15	800	0
			4	20	250	0
Superspeed	Cercon xt ML	CS	1	120	890	0
			2	30	1100	0
	Lava Esthetic	LS	3	15	1350	0
			4	10	1525	35
			5	120	750	0

**Table 3:** Three-way analysis of variance of translucency parameter results.

Source of Variation	Type III Sum of Squares	*df*	Mean Square	F	*p*
Corrected model	9.662 *	15	0.644	17.269	<0.001
Intercept	6296.813	1	6296.813	16,8812.541	<0.001
Z	6.125 × 10^−5^	1	6.125 × 10^−5^	0.002	0.968
S	4.046	1	4.046	108.457	<0.001
Layer	1.919	3	0.640	17.152	<0.001
Z × S	0.035	1	0.035	0.935	0.337
Z × Layer	3.260	3	1.087	29.134	<0.001
S × Layer	0.210	3	0.070	1.877	0.142
Z × S × Layer	0.192	3	0.064	1.716	0.172
Total	6308.862	80			
* R squared = 0.802 (Adjusted R squared = 0.755)					

S, sintering speed; Z, zirconia type.

**Table 4:** Translucency parameter for each group of the Cercon xt ML and Lava esthetic specimens (Mean ± standard deviation).

Code/Layer	EL	TL1	TL2	DL
**CC**	8.66 ^Ba^ (0.15)	8.92 ^Ba^ (0.14)	9.29 ^Cb^ (0.24)	9.60 ^Bb^ (0.18)
**CS**	8.17 ^Aa^ (0.18)	8.54 ^Ab^ (0.11)	8.69 ^Abb^ (0.20)	9.12 ^Ac^ (0.21)
LC *	9.33 ^Cb^ (0.14)	9.02 ^Bab^ (0.18)	9.02 ^Bcab^ (0.18)	8.93 ^Aa^ (0.30)
LS *	8.73 ^Ba^ (0.14)	8.56 ^Aa^ (0.22)	8.56 ^Aa^ (0.22)	8.83 ^Aa^ (0.22)

* Data for TL of Lava esthetic were used twice (TL1 and TL2) to compare with TL1 and TL2 of Cercon xt ML. The letters used to indicate statistical significance are lowercase for the layers and uppercase for the specimens on the vertical axes. Using the same letter implies the absence of a statistically significant difference.

**Table 5:** Three-way analysis of variance of opalescence parameter results.

Source of Variation	Type III Sum of Squares	*df*	Mean Square	F	*p*
Corrected model	177.777 *	13	13.675	42.388	<0.001
Intercept	17,857.593	1	17,857.593	55,351.475	<0.001
Z	3.541	1	3.541	10.975	0.002
S	41.047	1	41.047	127.231	<0.001
Layer	122.461	3	40.820	126.527	<0.001
Z × S	2.923	1	2.923	9.062	0.004
Z × Layer	0.445	2	0.223	0.690	0.506
S × Layer	3.512	3	1.171	3.628	0.018
Z × S × Layer	0.384	2	0.192	0.594	0.555
Total	18,805.572	70			
* R squared = 0.908 (Adjusted R squared = 0.886)					

S, sintering speed; Z, zirconia type.

**Table 6:** Opalescence parameter for each group of the Cercon xt ML and Lava esthetic specimens (Mean ± standard deviation).

Code/Layer	EL	TL1	TL2	DL
**CC**	15.22 ^Ba^ (0.22)	16.71 ^Bb^ (0.60)	17.47 ^Bc^ (0.29)	19.14 ^Bd^ (0.37)
**CS**	14.70 ^Ba^ (0.24)	15.60 ^Abb^ (0.25)	16.44 ^Bc^ (0.41)	17.27 ^Ad^ (0.25)
LC *	15.07 ^Ba^ (0.46)	16.73 ^Bb^ (1.06)	16.73 ^Bb^ (1.06)	19.14 ^Bc^ (0.60)
LS *	13.66 ^Aa^ (0.91)	14.34 ^Aa^ (0.86)	14.34 ^Aa^ (0.86)	16.78 ^Ab^ (0.53)

* Data for TL of Lava esthetic were used twice (TL1 and TL2) to compare with TL1 and TL2 of Cercon xt ML. The letters used to indicate statistical significance are lowercase for the layers and uppercase for the specimens on the vertical axes. Using the same letter implies the absence of a statistically significant difference.

**Table 7:** Differences in a* (Δa*) and b* (Δb*) between the reflected and transmitted colors (Mean ± standard deviation).

Code/Layer		EL	TL1	TL2	DL
Δa*	**CC**	1.17 (0.07) ^Ba^	1.45 (0.16) ^Bb^	1.56 (0.20) ^Bb^	2.08 (0.10) ^Bc^
	**CS**	0.67 (0.06) ^Aa^	0.97 (0.09) ^Ab^	1.30 (0.09) ^Ac^	1.77 (0.08) ^Ad^
	LC **	1.96 (0.14) ^Da^	2.29 (0.11) ^Db^	2.29 (0.11) ^Cb^	2.95 (0.16) ^Cc^
	LS **	1.63 (0.09) ^Ca^	1.77 (0.11) ^Ca^	1.77 (0.11) ^Ba^	2.23 (0.18) ^Bb^
Δb*	**CC**	15.18 (0.22) ^Ba^	16.55 (0.75) ^Bb^	17.40 (0.28) ^Bc^	19.02 (0.36) ^Bd^
	**CS**	14.68 (0.24) ^Ba^	15.57 (0.25) ^ABb^	16.39 (0.42) ^Bc^	17.18 (0.25) ^Ad^
	LC **	14.94 (0.45) ^Ba^	16.57 (1.06) ^Bb^	16.57 (1.06) ^Bb^	18.91 (0.58) ^Bc^
	LS **	13.57 (0.91) ^Aa^	14.23 (0.88) ^Aa^	14.23 (0.88) ^Aa^	16.63 (0.52) ^Ab^

** Data for TL of Lava esthetic were used twice (TL1 and TL2) to compare with TL1 and TL2 of Cercon xt ML. The letters used to indicate statistical significance are lowercase for the layers and uppercase for the specimens on the vertical axes. Using the same letter implies the absence of a statistically significant difference.

**Table 8:** Grain size for each group of the Cercon xt ML and Lava esthetic specimens (Mean ± SD).

Code/Layer		EL	DL
**Grain size (** **μm)**	**CC**	1.127 ^Ba^ (0.109)	1.149 ^BCa^ (0.099)
	**CS**	1.057 ^Aba^ (0.107)	1.093 ^Aba^ (0.092)
	**LC**	1.176 ^Ba^ (0.168)	1.228 ^Ca^ (0.090)
	**LS**	1.006 ^Aa^ (0.066)	1.001 ^Aa^ (0.120)

The letters used to indicate statistical significance are lowercase for the layers and uppercase for the specimens on the vertical axes. Using the same letter implies the absence of a statistically significant difference.

**Table 9:** Rietveld refinement results of X-ray diffraction data.

Layer		EL				DL			
Code		CC	CS	LC	LS	CC	CS	LC	LS
**Rwp (%)**		4.9632	4.3094	4.5387	4.4410	4.9479	4.1987	4.6400	4.4949
**Yttria-rich (*t*’)**	wt%	56.41 (35)	53.06 (46)	56.80 (32)	54.31 (31)	56.67 (34)	55.06 (47)	54.03 (28)	53.49 (24)
	*a* (Å)	3.6251	3.6239	3.6247	3.6239	3.6247	3.6238	3.6243	3.6236
	*c* (Å)	5.1514	5.1532	5.1515	5.1529	5.1512	5.1532	5.1515	5.1528
	*c/a*√2	1.0048	1.0055	1.0050	1.0055	1.0049	1.0055	1.0051	1.0055
	Y_2_O_3_ (mol%)	7.0944	6.7979	7.0378	6.8232	7.0632	6.7859	6.9896	6.7957
**Yttria-lean (*t*)**	wt%	43.58 (35)	46.93 (46)	43.19 (32)	45.68 (31)	43.32 (34)	44.93 (47)	45.96 (28)	46.50 (24)
	*a* (Å)	3.6079	3.6088	3.6070	3.6082	3.6071	3.6085	3.6064	3.6079
	*c* (Å)	5.1791	5.1770	5.1801	5.1778	5.1788	5.1776	5.1797	5.1779
	*c/a*√2	1.0150	1.0144	1.0155	1.0147	1.0152	1.0146	1.0156	1.0148
	Y_2_O_3_ (mol%)	2.8284	3.0955	2.6486	2.9645	2.7618	3.0142	2.6125	2.9228

The values in parentheses represent the estimated standard deviation in the least significant figure to the left.

## Data Availability

Not applicable.
